# Association of Potentially Inappropriate Medications and Geriatric Nutritional Risk Index with Frailty in Elderly Patients with Ischemic Heart Disease

**DOI:** 10.3390/diagnostics16132094

**Published:** 2026-07-03

**Authors:** Pei-Ru Lin, Chew-Teng Kor, Yu-Chung Wei, Yen-Tze Liu

**Affiliations:** 1 Big Data and Digital AI Application Center, Changhua Christian Hospital, Changhua 500, Taiwan; 183778@cch.org.tw (P.-R.L.); 179297@cch.org.tw (C.-T.K.); 2 Department of Mathematics, National Changhua University of Education, Changhua 500, Taiwan; 3 Graduate Institute of Clinical Medicine, College of Medicine, National Chung Hsing University, Taichung 402, Taiwan; 4 Graduate Institute of Statistics and Information Science, National Changhua University of Education, Changhua 500, Taiwan; weiyuchung@cc.ncue.edu.tw; 5 Department of Family Medicine, Changhua Christian Hospital, Changhua 500, Taiwan; 6 Department of Post-Baccalaureate Medicine, College of Medicine, National Chung Hsing University, Taichung 402, Taiwan

**Keywords:** potentially inappropriate medications, geriatric nutritional risk index, frailty, ischemic heart disease

## Abstract

**Background/Objectives**: Frailty is a clinically significant syndrome in older patients with ischemic heart disease, associated with adverse outcomes including hospitalization, disability, and mortality. This study aimed to evaluate the association of potentially inappropriate medication (PIM) and the Geriatric Nutrition Risk Index (GNRI) with the incidence of frailty among elderly patients with ischemic heart disease (IHD). **Methods**: This retrospective cohort study enrolled elderly patients with IHD between January 2018 and March 2024. Patients were grouped by PIM use and GNRI levels (<92 vs. ≥92). Cox proportional hazards models assessed the associations of PIM and GNRI with the incidence of frailty. Subgroup and sensitivity analyses evaluated the consistency and robustness of these findings. **Results**: PIM use was associated with a significantly higher risk of frailty (HR = 3.01, 95% CI = 2.48–3.65) than non-use. Similarly, lower GNRI increased the risk of frailty compared to higher GNRI (HR = 1.31, 95% CI = 1.12–1.54). Patients with both PIM and lower GNRI may have a higher risk of frailty, with an adjusted aHR of 4.09. Subgroup analyses showed significant interactions between GNRI and hypertension. Sensitivity analyses indicated that PIM (aHR = 2.65) and lower GNRI (aHR = 1.14) remained significantly associated with frailty, even after including those with pre-existing frailty. **Conclusions**: For elderly patients with IHD, both PIM and lower GNRI were significantly associated with the incidence of frailty. These findings suggest that PIM exposure and low GNRI may serve as clinically accessible markers for identifying older IHD patients at elevated risk of frailty.

## 1. Introduction

With the continuous advancement of healthcare and improvements in quality of life, the global population is undergoing a rapid demographic shift toward aging. By 2039, individuals aged 65 years and older are projected to constitute 14% of the global population, marking the transition into an aged society [1]. Taiwan, in particular, is expected to become a super-aged society by 2027, with 21.8% of its population aged 65 years or older, representing one of the fastest aging populations worldwide [2]. Among the elderly, cardiovascular disease (CVD) remains one of the most prevalent chronic diseases and is the leading cause of mortality worldwide [3]. Ischemic heart disease (IHD) accounts for approximately 37.7% of all CVD, making it not only the most common subtype but also a significant contributor to cardiovascular-related deaths [4].

Frailty is a common clinical syndrome in the elderly, characterized by diminished physiological reserves and reduced capacity to respond to external stressors such as illness or injury. This condition often results from the cumulative decline of multiple physiological systems associated with aging and chronic disease [5]. A previous systematic review reported a pooled frailty prevalence of 19% among individuals with IHD (95% CI, 15–24%), although estimates across studies ranged widely from 4% to 61% [6]. Frailty in this population has been strongly associated with adverse outcomes, including higher all-cause mortality, increased hospital readmission rates, and a greater incidence of major adverse cardiovascular events [7,8].

Beyond the underlying disease itself, medication use and nutritional status are critical factors influencing frailty in the elderly. Many medications pose a higher risk of adverse effects in the elderly and have been identified as requiring caution or avoidance; these are referred to as potentially inappropriate medications (PIMs) [9]. While evidence regarding the impact of PIMs on frailty in patients with IHD remains limited, previous studies have demonstrated the risks of critical PIM classes. Central nervous system-active medications are associated with falls, which may contribute to frailty-related vulnerability [10], while exposure to anticholinergic drugs is associated with a higher occurrence of frailty [11]. These medications may contribute to functional decline by disrupting electrolyte balance, impairing neuromuscular function, reducing nutrient absorption, and affecting bone health, thereby increasing vulnerability to frailty, falls, hospitalization, and death.

Malnutrition is another key contributor to the onset and progression of frailty. Nutritional assessment is essential in geriatric care, and the Geriatric Nutritional Risk Index (GNRI) has been widely adopted due to its simplicity and prognostic value [12,13]. While lower GNRI scores have been associated with increased mortality risk in various chronic diseases, including acute coronary syndrome [14], research examining the relationship between GNRI and frailty in IHD patients remains limited.

Although both PIMs and malnutrition have been associated with frailty, few studies have evaluated their combined effects in elderly patients with IHD. Therefore, this study aims to comprehensively evaluate the influence of PIM and GNRI on frailty among patients with IHD. By examining the interplay between pharmacological and nutritional risks, the findings are expected to inform clinical strategies for early identification and intervention, and to support the development of integrated care approaches that improve health outcomes in this vulnerable population.

## 2. Materials and Methods

### 2.1. Study Population

**Figure 1:**
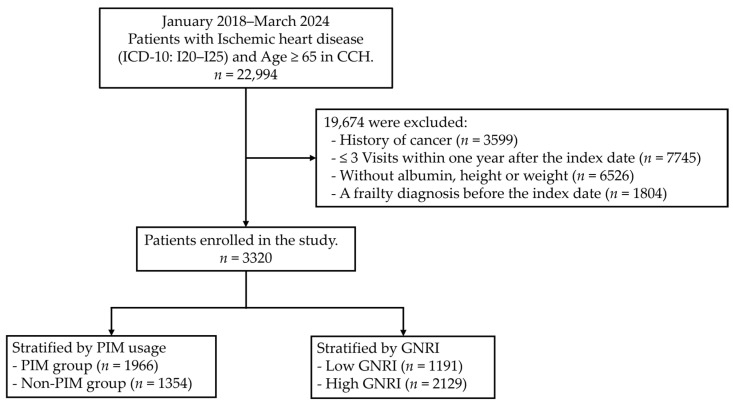
Flow chart of study patients. Low GNRI was defined as GNRI < 92; High GNRI was defined as GNRI ≥ 92. Abbreviations: CCH, Changhua Christian Hospital; PIM, potentially inappropriate medication; GNRI, Geriatric Nutritional Risk Index.

This retrospective cohort study included patients aged ≥65 years diagnosed with IHD (ICD-10: I20–I25) between January 2018 and March 2024 at Changhua Christian Hospital (CCH), a tertiary medical center located in central Taiwan. Patients were categorized based on their exposure to PIMs. The index date was defined as the date of first use of any medication classified as a PIM. The following exclusion criteria were applied: (1) history of cancer, (2) three or fewer outpatient visits within one year after the index date, (3) without serum albumin, height or body weight measurements, and (4) a diagnosis of frailty before the index date. A total of 3320 eligible patients were included in the final analysis (Figure 1). The endpoint of this study was incident frailty. Patient data were censored at the date of death, the last available follow-up date, or the end of the 1-year follow-up period, whichever occurred first. The Institutional Review Board of CCH granted a waiver for the informed consent requirement and approved this study (IRB No: 250521).

This study extracted patient information from the CCH clinical research database, including demographics (age, gender, height, and weight), laboratory data, comorbidities (cerebrovascular accident, hypertension, diabetes mellitus, hyperlipidemia, renal disease, and chronic obstructive pulmonary disease (COPD)), and the Charlson Comorbidity Index (CCI). All data were obtained from the last clinical visit prior to the index date.

### 2.2. Definition of Potentially Inappropriate Medication

PIMs were identified based on the 2023 update of the Beers Criteria [9]. The Beers Criteria, originally developed by Mark Beers, MD, identifies medications whose potential harms often outweigh their benefits in older adults and are best avoided under most circumstances. High-risk medications include immediate-release antihypertensive agents (e.g., nifedipine), anticoagulants (e.g., warfarin), and drugs with strong anticholinergic properties (e.g., certain antidepressants and antiparkinsonian agents), as well as benzodiazepines, opioids, tricyclic antidepressants, and antipsychotics.

Participants were considered to have PIM exposure if they had continuously used any of the listed medications for more than three months. The three-month criterion was chosen to better reflect chronic medication exposure, which is more likely to impact long-term health outcomes in older adults. For these PIM-exposed patients, the index date was defined as the date of their initial PIM prescription. For non-PIM patients, the index date was assigned as a randomly selected qualifying outpatient visit after the first IHD diagnosis during the study period.

### 2.3. Geriatric Nutritional Risk Index (GNRI) Measurement and Classification

The GNRI was used to assess nutritional risk, calculated as 1.489 × serum albumin (g/L) + 41.7 × (present weight/ideal weight) [13], where the ideal weight [15] was defined as 22 × (height (m))^2^. If the present weight exceeded the ideal weight, the ratio was set to 1.0.

Previous studies have reported that a GNRI < 92 is associated with higher mortality in elderly patients with heart failure [16,17]. In addition, other studies have utilized the same cutpoint to demonstrate an association with a higher incidence of fractures [18,19]. Therefore, this study selected 92 as a pre-specified threshold. Accordingly, patients were classified into the high GNRI group (GNRI ≥ 92), indicating better nutritional status, and the low GNRI group (GNRI < 92), indicating poorer nutritional status.

### 2.4. Frailty Assessment

Frailty is a critical and widely recognized indicator for assessing the health status of older adults, particularly their ability to manage and recover from diseases, injuries, or medical treatments. This study utilizes the multimorbidity frailty index (mFI) method, developed by Hsi-Yu Lai et al. based on Taiwan National Health Insurance data for people over 65 years old [20]. This index quantifies the degree of individual frailty through 38 ICD-10-CM diagnostic code items. The mFI score is calculated using the following formula: mFI-v10 = number of deficits a patient has/number of total deficits items. To be defined as having the defect, a patient had to have at least three outpatient claims or one inpatient claim for that specific diagnosis code. The mFI has demonstrated good validity and applicability across various clinical settings. Its predictive performance has been specifically validated in populations with diabetes [21,22], heart failure [23,24], prostate cancer [25], and among general surgical patients [26]. In this study, referring to the method of Hsi-Yu Lai et al. [23], a mFI-v10 score exceeds 0.105 is used as the definition of frailty to distinguish between fit and frail groups.

### 2.5. Statistical Analysis

Categorical variables were presented as number and percentage, and continuous variables were presented as medians with interquartile ranges (IQR). Differences between groups were compared using the chi-square test for categorical variables and Mann–Whitney U test for continuous variables. Separate Kaplan–Meier (KM) curves were generated to evaluate the impact of PIM and GNRI categories on frailty incidence, with comparisons performed using the log-rank test. The crude and multivariable Cox proportional hazards models were utilized to assess the association between PIM status, GNRI levels, and frailty. The findings were presented as hazard ratios (HR) with 95% confidence intervals (CI).

Subgroup analyses were performed at two levels: (1) by stratifying the cohort by PIM status to assess whether the association between GNRI and frailty differed by PIM group; and (2) by stratifying by clinical and laboratory subgroups (e.g., age, triglycerides (TG) level, estimated glomerular filtration rate (eGFR) level, hypertension, COPD), with both PIM group and GNRI category included simultaneously in multivariable models to examine effect modification by these factors. The results were visualized using a forest plot, which presented the HR and their corresponding 95% CI for each subgroup. In the forest plot, the vertical line represents the null value (HR = 1), while the point estimates and confidence intervals illustrate the differences in effect sizes. This visualization provides a straightforward and intuitive method to assess the consistency or heterogeneity of the associations and to identify any significant interactions.

To enhance the robustness of our findings, several sensitivity analyses were performed to re-evaluate the associations of PIM and GNRI with frailty: (1) inclusion of individuals with frailty before the index date; (2) performing Fine and Gray subdistribution hazards model to account for death as a competing event; (3) exclusion of individuals with a follow-up time of ≤3 months to address potential immortal time bias; (4) exclusion of patients with specific comorbidities, including cerebrovascular accident, hypertension, renal disease, and COPD; and (5) implementation of stricter criteria for frailty to address potential incorporation bias. Specifically, to prevent the double-counting of overlapping baseline diagnostic codes (Table S1), if a patient had a specific comorbidity at baseline, the corresponding overlapping ICD codes were not counted toward their frailty outcome during the follow-up period. All statistical analyses were performed using SAS software, version 9.4 (SAS Institute Inc., Cary, NC, USA). The forest plot for subgroup and interaction analyses was generated using R software, version 4.5.2 (R Foundation for Statistical Computing, Vienna, Austria; available at http://cran.r-project.org (accessed on 21 November 2025)). A two-sided *p*-value < 0.05 was considered statistically significant.

## 3. Results

### 3.1. Baseline Characteristics

A total of 3320 elderly patients with ischemic heart disease were included in the study. During the cumulative follow-up period of 32,033 person-months, incident frailty was observed in 744 patients (22.4%). The median follow-up period was 12 months (IQR, 7.4–12 months). The median age of the cohort was 74 years (IQR, 68–81 years), and 1898 participants (57.2%) were male. Of the total population, 1966 patients (59.2%) were classified into the PIM group, while 1354 patients (40.8%) were classified into the non-PIM group. Within the PIM group, a total of 11,580 prescription records were identified (Table S2). The most frequently prescribed drug classes were sedative-hypnotics (*n* = 3249; 28.06%), proton pump inhibitors (*n* = 3032; 26.18%), and cardiovascular drugs (*n* = 2023; 17.47%). Together, these three classes accounted for more than 70% of all PIM records. Among the 1966 PIM users, sedative-hypnotics remained the most common exposure (*n* = 924; 47.00%), followed closely by proton pump inhibitors (*n* = 910; 46.29%) and cardiovascular drugs (*n* = 626; 31.84%). The median GNRI among patients was 95.3 (IQR = 87.86–99.77). Patients were divided into low and high GNRI group according to the cutoff value, of which 1191 patients (35.9%) were classified into the low GNRI group and 2129 patients (64.1%) were classified into the high GNRI group. According to the mFI, 744 patients (22.4%) were identified as frail, and 2576 patients (77.6%) were considered non-frail. The demographic and baseline clinical characteristics of all participants are presented in Table 1.

Comparing the PIM and non-PIM groups, there was no significant differences in age distribution, with the median age of 74 years for both groups. Approximately 60% of participants in the non-PIM group were male. The PIM group had higher levels of total cholesterol and low-density lipoprotein (LDL) cholesterol, while the non-PIM group had a higher incidence of hyperlipidemia and renal disease. There were no statistical differences in other variables between the two groups. Moreover, the prevalence of frailty was notably higher among patients in the PIM group than among those in the non-PIM group (30.4% vs. 10.9%).

Differences were also noted between the high and low GNRI groups. Patients in the low GNRI group were older (median age: 77 vs. 72 years) and had higher CCI scores (median: 5 vs. 4) compared with those in the high GNRI group. However, the proportion of male patients was higher in the high GNRI group (58.8%). Laboratory findings revealed that the low GNRI group had significantly lower levels of hemoglobin (Hb), total cholesterol, high-density lipoprotein (HDL) cholesterol, TG, eGFR, and glutamate pyruvate transaminase (GPT), along with higher white blood cell (WBC) counts. The low GNRI group had a higher prevalence of cerebrovascular accident, diabetes, renal disease, and COPD, but a lower prevalence of hyperlipidemia. Furthermore, the low GNRI group had a higher prevalence of frailty than the high GNRI group (27% vs. 19.9%).

### 3.2. Association of PIM and Nutritional Status with Frailty Risk

**Figure 2:**
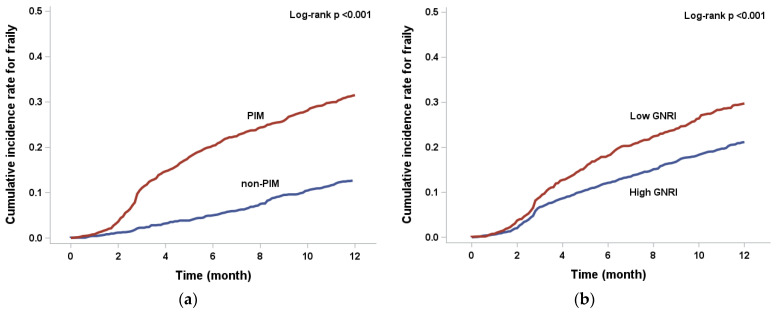
Kaplan–Meier curves for frailty by (**a**) Potentially inappropriate medication (PIM) (**b**) geriatric nutritional risk index (GNRI).

The Kaplan–Meier plot of the cumulative incidence of frailty at one year is shown in Figure 2, illustrating the cumulative incidence rates stratified by PIM and GNRI groups. Compared to the non-PIM group, elderly patients in the PIM group demonstrated a significantly higher incidence of frailty (log-rank *p* < 0.001; Figure 2a). The mean time to frailty development was shorter in the PIM group than in the non-PIM group (9.87 vs. 11.33 months). Similarly, patients in the low GNRI group exhibited a higher incidence of frailty compared to those in the high GNRI group (log-rank *p* < 0.001; Figure 2b).

Cox proportional hazards models were used to evaluate the associations between PIM, GNRI levels, and the risk of frailty (Table 2). In the unadjusted model, compared with the non-PIM group, patients in the PIM group were associated with an increased risk of frailty (HR = 2.95, 95% CI = 2.46–3.53, *p* < 0.001). Similarly, patients in the low GNRI group exhibited were associated with an increased risk of frailty compared to those in the high GNRI group (HR = 1.49, 95% CI = 1.29–1.73, *p* < 0.001). In the multivariable analysis using backward selection, age, TG, eGFR, HTN, and COPD were retained in the final model. After adjusting for these variables, PIM was significantly associated with higher rates of frailty (aHR = 3.01, 95% CI = 2.48–3.65, *p* < 0.001), and lower GNRI was also significantly associated with frailty (aHR = 1.31, 95% CI = 1.12–1.54, *p* < 0.001).

**Figure 3:**
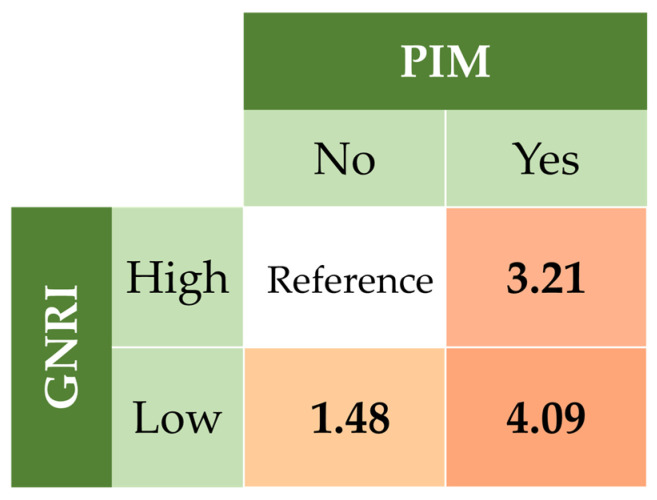
Adjusted hazard ratios for incident frailty according to combined PIM and GNRI categories. The high-GNRI/non-PIM group served as the reference group. Cells with HRs > 1.0 are shaded in orange from light to dark according to the magnitude of the HR. Bold numbers indicate statistical significance at *p* < 0.05. Abbreviations: PIM, potentially inappropriate medication; GNRI, Geriatric Nutritional Risk Index.

**Figure 4:**
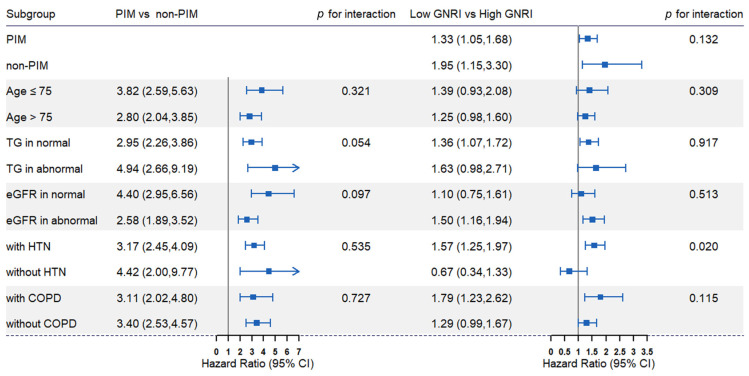
Subgroup analysis of the prognostic impact of PIM and GNRI: evaluating interactions across Age, TG, eGFR, Hypertension, and COPD. TG normal range: <150 mg/dL; eGFR normal range: >60 mL/min/1.73 m^2^. Abbreviations: PIM, potentially inappropriate medication; GNRI, Geriatric Nutritional Risk Index; TG, triglycerides; eGFR, estimated glomerular filtration rate; HTN, hypertension; COPD, chronic obstructive pulmonary disease.

Figure 3 presents the frailty risk matrix determined by PIM and GNRI levels. Using patients with high GNRI and non-PIM as the reference group, those with low GNRI but non-PIM had an increased risk of frailty (aHR = 1.48, 95% CI = 1.04–2.11). Patients with high GNRI but PIM had a higher risk (aHR = 3.21, 95% CI = 2.47–4.16). The highest risk was observed among patients with both lower GNRI and PIM, with an aHR of 4.09 (95% CI = 3.12–5.35). Although, a stepwise increase in frailty risk was observed across PIM and GNRI categories, there was no significant interaction (*p* for interaction = 0.132, Figure 4), suggesting no statistical evidence of a multiplicative interaction between PIM and GNRI in this cohort.

### 3.3. Subgroup Analysis

When PIM and GNRI category were simultaneously included in multivariable models within each clinical subgroup, there were no significant interactions in most subgroups. In addition, we found a significant interaction between hypertension and GNRI (*p* for interaction = 0.020). Among patients with hypertension (*n* = 2611; frailty events = 634), lower GNRI was significantly associated with the risk of frailty (aHR = 1.57; 95% CI = 1.25–1.97). In contrast, in the smaller subgroup without hypertension (*n* = 709; frailty events = 110), the association was not statistically significant (aHR = 0.67; 95% CI = 0.34–1.33).

### 3.4. Sensitivity Analysis

To evaluate the robustness of our primary findings, we performed a series of sensitivity analyses (Table 3). First, when including individuals with a frailty diagnosis before the index date (*n* = 5124), the associations of PIM (aHR = 2.65; 95% CI = 2.35–2.99, *p* < 0.001) and low GNRI (aHR = 1.14; 95% CI = 1.03–1.26, *p* = 0.010) with frailty remained statistically significant, although the estimates were slightly attenuated compared with the primary analysis. Second, the results were highly consistent in a competing risk analysis, with an aHR of 3.05 (95% CI = 2.52–3.69, *p* < 0.001) for PIM and 1.30 (95% CI = 1.11–1.52, *p* = 0.001) for low GNRI. Third, excluding individuals with a follow-up time of ≤3 months (*n* = 2990) did not alter the significant associations for either PIM (aHR = 2.41; 95% CI = 1.93–3.01, *p* < 0.001) or low GNRI (aHR = 1.38; 95% CI = 1.14–1.68, *p* < 0.001). Fourth, we restricted the cohort by excluding individuals with specific comorbidities, including cerebrovascular accident, hypertension, renal disease, and COPD (*n* = 338). In this population, the risk associated with PIM remained significant (aHR = 2.48; 95% CI = 1.03–5.98, *p* = 0.043); while the association between low GNRI and frailty was no longer statistically significant (aHR = 1.19; 95% CI = 0.60–2.36, *p* = 0.628). Finally, to address the potential incorporation bias, we adopted stricter criteria for the frailty outcome that prevented the double-counting of overlapping baseline codes. This restriction reduced the total number of identified frailty events from 744 to 330. Under these stricter criteria, the adjusted HR for PIM increased from 3.01 to 5.40 (95% CI = 3.74–7.80, *p* < 0.001) for the risk of frailty, and the association between low GNRI and frailty remained significant (aHR = 1.35; 95% CI = 1.06–1.71, *p* = 0.015).

## 4. Discussion

This study investigated the effects of PIMs and malnutrition, as measured by the GNRI, on the incidence of frailty in elderly patients with IHD. The findings revealed that both PIM and lower GNRI levels were significant and risk factors for frailty. No significant interaction was observed between the two, suggesting that their contributions to frailty may occur through distinct underlying mechanisms.

Recent studies have increasingly recognized frailty not as a single disease, but as a multifactorial clinical syndrome resulting from complex interactions among genetic, endocrine, chronic inflammatory, musculoskeletal, and psychosocial factors [27]. Patients with IHD often experience chronic low-grade inflammation, characterized by elevated levels of interleukin-6 (IL-6) and C-reactive protein (CRP), which have been associated with sarcopenia, physical decline and frailty [28]. This study found that the prevalence of frailty in elderly patients with IHD was 22.4%, which is consistent with the 15–24% prevalence reported in previous studies [6], supporting the classification of IHD patients as a high-risk population for frailty.

The results of this study showed that PIMs had a significantly higher risk of frailty than those without, particularly in the elderly patients with IHD. Among all PIM prescriptions, sedative-hypnotics (e.g., benzodiazepines), proton pump inhibitors (PPIs), and cardiovascular drugs (e.g., dipyridamole, alpha-blockers) were the three most frequently prescribed PIM classes. Notably, the first two classes each accounted for more than 20% of all PIM records and affected over 45% of all PIM-exposed patients. Given the high frequency of these medications, our discussion focuses on their association with frailty. Given the inherent limitations of our retrospective observational design, this study could not directly explore the underlying pharmacological mechanisms; therefore, we propose several potential pathways based on the existing literature to help explain this observed association. Sedative and hypnotic medications may suppress central nervous system activity, which can lead to confusion, drowsiness, and cognitive impairment [29]. These effects may reduce engagement in daily activities and mobility, increase the risk of falls, and contribute to the progression of muscle atrophy [10]. Dipyridamole, an antiplatelet drug, may cause dizziness and syncope [30]. Studies have shown that antihypertensive agents and other cardiovascular medications were associated with an increased risk of orthostatic hypotension (OH) [31], which may increase the likelihood of adverse outcomes such as stroke, falls, cognitive decline, and mortality [32,33,34]. Moreover, previous studies have demonstrated a significant association between OH and frailty in the elderly [35]. Alpha-blockers, found in some antihypertensive agents, may indirectly reduce bone mineral density (BMD) [36], increasing the risk of fractures and ischemic stroke [37,38]. Furthermore, long-term use of PPIs was associated with a variety of adverse effects, including renal disease, stroke, fractures, and dementia [39,40,41,42]. The cumulative effects of these medications on the neurocognitive and musculoskeletal systems may play a significant role in the development of frailty in older adults.

On the other hand, nutritional status has also been identified as a key factor influencing the development of frailty [43]. In this study, the GNRI was used to assess nutritional status in older adults. The GNRI incorporates serum albumin and ideal body weight, serving as an indicator of protein nutritional status and body weight changes. In this study, we adopted a pre-specified GNRI threshold of 92, based on previous research, and our study supplements the impact of GNRI on frailty results. When nutritional intake is inadequate, especially protein deficiency, it may impair immune function and promote the release of inflammatory cytokines, such as IL-6 and TNF-α, inducing chronic low-grade inflammatory response [44], which in turn stimulates muscle protein catabolism and accelerates the loss of muscle mass and strength. Previous studies have demonstrated a linear relationship between lower serum albumin and decreased handgrip strength [44]. Lower serum albumin has been associated with frailty and reduced muscle strength in older patients with chronic coronary syndrome. GNRI is also closely related to bone metabolism [45]. It has been positively associated with BMD, and individuals with lower GNRI scores are at increased risk of osteoporosis, thereby elevating the likelihood of falls and fractures [46]. Overall, the GNRI reflects not only the nutritional status of older adults but may also assesses frailty risk through multiple pathways, including inflammation, muscle health, and BMD.

The association between low GNRI and incident frailty was attenuated after adjustment, likely reflecting the substantial baseline differences between GNRI groups, including age, comorbidity burden, renal function, and hemoglobin levels. In contrast, the PIM estimate changed little after adjustment, suggesting that the measured covariates retained in the final model did not materially confound the observed PIM–frailty association in this dataset. Nevertheless, this finding should not be interpreted as evidence of a causal or isolated effect of PIM exposure, and residual confounding from unmeasured factors remains possible. Furthermore, through a series of sensitivity analyses, the primary associations remained significant. In the sub-cohort excluding individuals with specific baseline comorbidities, the association between GNRI and frailty was not statistically significant, which may be due to the smaller sample size leading to reduced statistical power. Overall, these sensitivity analyses showed that our primary findings are robust and reliable.

Moreover, our subgroup analysis showed a significant interaction between hypertension and GNRI status, indicating that the effect of nutritional status on frailty risk differed by hypertension status. A lower GNRI was significantly associated with the risk of frailty in hypertensive patients. This finding is consistent with existing research on hypertensive populations, which has shown that a lower GNRI is associated with an increased incidence of stroke [47] and other adverse events, including renal events, cardiovascular events, and all-cause mortality [48]. Mechanistically, these studies suggest that a lower GNRI reflects a status of protein depletion, which can lead to increased oxidative stress and systemic inflammation [47,48]. Furthermore, CVD patients typically present with a high prevalence of hypertension; whereas, non-hypertensive CVD cases are relatively rare. This clinical distribution suggests that, within our restricted analysis, the smaller sample size of this specific subgroup may have led to reduced statistical power, which perhaps explains why the association did not reach statistical significance.

In summary, this study highlights that the PIM and poor nutritional status are both significant risk factors for the development of frailty among elderly patients with IHD. Adverse effects related to medications may contribute to functional decline through multiple physiological mechanisms, with central nervous system suppression representing may be a key neurofunctional pathway, alongside other systemic effects such as bone metabolism disruption, hypotension, and increased infection risk. Additionally, inadequate nutrition may exacerbate symptoms of frailty by compromising muscle mass and immune reserves. Therefore, clinical practice should pay more attention to the prescription profiles and nutritional assessments of elderly patients with IHD. By early identification and adjustment of inappropriate medications, alongside timely nutritional interventions, may help slow the progression of frailty and improve the patient’s quality of life and clinical outcomes. Future research can explore the efficacy of specific pharmacological or nutritional interventions to establish more evidence-based strategies for geriatric care.

However, this study also has some limitations. First, this study is a retrospective observational study, and causality could not be determined. Second, the findings of this single-center study in Taiwan may not be fully generalizable to other populations. Third, both PIM and GNRI were measured at a single time point, which may not capture long-term medication patterns or changes in nutritional status, potentially underestimating their dynamic effects on the development of frailty. Furthermore, the use of PIMs in these patients may reflect underlying clinical vulnerabilities. Patients receiving these medications often have more comorbidities or poorer baseline functional status, which in themselves may increase the risk of frailty. Although multivariable models were utilized to adjust for these factors, residual confounding remains possible in this observational study. Finally, this study may not have fully captured all potential factors contributing to the development of frailty. While the analysis primarily focused on pharmacological and nutritional aspects, frailty is a multifactorial condition that may also be influenced by other variables such as psychological status (e.g., depression), living arrangements, physical activity levels, and social determinants [49].

## 5. Conclusions

This study demonstrates that PIM and lower GNRI were both significantly associated with the incidence of frailty in older adults with IHD. These findings suggest that PIM exposure and low GNRI may serve as clinically accessible markers for identifying older IHD patients at elevated risk of frailty. Prospective studies are needed to determine whether medication optimization or nutritional interventions can reduce frailty development.

## Figures and Tables

**Table 1:** Baseline characteristics of the study population.

Variable	PIM	Non-PIM	*p*-Value	Low GNRI	High GNRI	*p*-Value
Sample Size	1966	1354		1191	2129	
Age	74 (68–81)	74 (68–81)	0.696	77 (70–84)	72 (68–80)	<0.001
Gender, Male	1087 (55.3%)	811 (59.9%)	0.008	646 (54.2%)	1252 (58.8%)	0.011
Charlson comorbidity index	4 (2–6)	4 (2–6)	0.012	5 (3–6)	4 (2–5)	<0.001
Comorbidity-*n* (%)						
Cerebrovascular accident	261 (13.3%)	156 (11.5%)	0.134	175 (14.7%)	242 (11.4%)	0.006
Hypertension	1534 (78.0%)	1077 (79.5%)	0.295	934 (78.4%)	1677 (78.8%)	0.295
Diabetes mellitus	1083 (55.1%)	766 (56.6%)	0.397	697 (58.5%)	1152 (54.1%)	0.014
Hyperlipidemia	1323 (67.3%)	956 (70.6%)	0.043	716 (60.1%)	1563 (73.4%)	<0.001
Renal disease	1131 (57.5%)	839 (62.0%)	0.011	758 (63.6%)	1212 (56.9%)	<0.001
Chronic Obstruction Pulmonary Disease	395 (20.1%)	293 (21.6%)	0.279	283 (23.8%)	405 (19.0%)	0.001
Laboratory data						
Hb, g/dL	12.5 (10.8–13.8)	12.6 (10.9–14.1)	0.090	11.35 (9.9–13)	13 (11.7–14.2)	<0.001
Total Cholesterol, mg/dL	148 (127–174)	143 (123–169)	<0.001	143 (122–171)	148 (128–172)	0.001
HDL-Cholesterol, mg/dL	41 (34–49)	41 (34–49.5)	0.716	40 (33–48)	42 (35–50)	<0.001
LDL-Cholesterol, mg/dL	81 (65–103)	78 (62–98)	<0.001	80 (64–101)	80 (64–100)	0.872
Triglyceride, mg/dL	110 (77–152)	107 (78–150)	0.623	104 (74–139)	113 (79–158)	<0.001
HbA1c, %	6.2 (5.7–7.1)	6.2 (5.7–7.1)	0.719	6.2 (5.7–7.2)	6.2 (5.7–7)	0.992
Creatinine, mg/dL	1.1 (0.8–1.5)	1.0 (0.8–1.5)	0.530	1.1 (0.8–2.0)	1.0 (0.8–1.4)	<0.001
eGFR, mL/min/1.73 m^2^	63.0 (41.7–82.3)	65.1 (40.6–85.5)	0.125	58.9 (30.3–79.9)	66.1 (46.1–84.8)	<0.001
Uric Acid, mg/dL	5.9 (4.9–7.2)	5.8 (4.9–7.1)	0.407	5.9 (4.8–7.2)	5.9 (5–7.1)	0.445
GOT, U/L	26 (21–34)	26 (21–33)	0.409	26 (21–35)	26 (22–33)	0.595
GPT, U/L	21 (15–30)	20 (14–29)	0.116	19 (14–29)	21 (15–30)	<0.001
WBC, 10^3^/μ	6.9 (5.6–8.8)	6.8 (5.6–8.4)	0.163	7.1 (5.7–9.1)	6.7 (5.5–8.3)	<0.001
K, mmol/L	4 (3.7–4.3)	4 (3.7–4.3)	0.729	4 (3.7–4.3)	4 (3.7–4.3)	0.853
Outcome after index date						
Frailty	597 (30.4%)	147 (10.9%)	<0.001	321 (27.0%)	423 (19.9%)	<0.001

Abbreviations: PIM, potentially inappropriate medication; GNRI, geriatric nutritional risk index; Hb, hemoglobin; HDL-Cholesterol, High-density lipoprotein cholesterol; LDL-Cholesterol, Low-density lipoprotein cholesterol; HbA1c, glycated hemoglobin; eGFR, estimated glomerular filtration rate; GOT, glutamic oxaloacetic transaminase; GPT, glutamic pyruvic transaminase; WBC, white blood cell.

**Table 2:** Cox regression model for frailty.

	Univariable Cox Regression		Multivariable Cox Regression	
	cHR (95% CI)	*p*-Value	aHR (95% CI) ^1^	*p*-Value
PIM vs. non-PIM	2.95 (2.46, 3.53)	<0.001	3.01 (2.48, 3.65)	<0.001
Low GNRI vs. high GNRI	1.49 (1.29, 1.73)	<0.001	1.31 (1.12, 1.54)	<0.001
Age	1.04 (1.03, 1.05)	<0.001	1.02 (1.01, 1.03)	<0.001
TG	0.999 (0.998, 1.00)	0.022	0.999 (0.998, 1.00)	0.017
eGFR	0.99 (0.99, 0.99)	<0.001	0.99 (0.99, 1.00)	<0.001
HTN	1.67 (1.36, 2.04)	<0.001	1.66 (1.32, 2.08)	<0.001
COPD	1.52 (1.29, 1.78)	<0.001	1.42 (1.19, 1.69)	<0.001

^1^ Adjusted for age, gender, comorbidities, and laboratory data with backward selection. Abbreviations: PIM, potentially inappropriate medication; TG, triglycerides; eGFR, estimated glomerular filtration rate; HTN, hypertension; COPD, chronic obstructive pulmonary disease; GNRI, geriatric nutritional risk index.

**Table 3:** Sensitivity analyses for the associations of potentially inappropriate medication (PIM) and Geriatric Nutritional Risk Index (GNRI) with incident frailty.

	Multivariable Cox Regression	
	aHR (95%CI)	*p*-Value
Main analysis (*n* = 3320)		
PIM vs. non-PIM	3.01 (2.48, 3.65)	<0.001
Low GNRI vs. high GNRI	1.31 (1.12, 1.54)	<0.001
Include individuals with a frailty diagnosis before the index date (*n* = 5124)		
PIM vs. non-PIM	2.65 (2.35, 2.99)	<0.001
Low GNRI vs. high GNRI	1.14 (1.03, 1.26)	0.010
Competing risk analysis ^1^ (*n* = 3320)		
PIM vs. non-PIM	3.05 (2.52, 3.69)	<0.001
Low GNRI vs. high GNRI	1.30 (1.11, 1.52)	0.001
Exclude individuals with a follow-up time of ≤3 months (*n* = 2990)		
PIM vs. non-PIM	2.41 (1.93, 3.01)	<0.001
Low GNRI vs. high GNRI	1.38 (1.14, 1.68)	<0.001
Exclude individuals with specific comorbidities ^2^ (*n* = 338)		
PIM vs. non-PIM	2.48 (1.03, 5.98)	0.043
Low GNRI vs. high GNRI	1.19 (0.60, 2.36)	0.628
Modified incident frailty definition ^3^ (*n* = 3320)		
PIM vs. non-PIM	5.40 (3.74, 7.80)	<0.001
Low GNRI vs. high GNRI	1.35 (1.06, 1.71)	0.015

^1^ Fine and Gray subdistribution hazards model was performed to account for death as a competing event. ^2^ Specific comorbidities: cerebrovascular accident (CVA), hypertension (HTN), renal disease, and chronic obstructive pulmonary disease (COPD). ^3^ Modified incident frailty definition: To address the potential incorporation bias, overlapping ICD-10-CM codes corresponding to specific baseline comorbidities (CVA, HTN, renal disease, and COPD) were not counted toward the frailty outcome during the follow-up period.

## Data Availability

The data that support the findings of this study originate from Changhua Christian Hospital clinical research database. Restrictions apply to the availability of these data and they are therefore not publicly available.

## Supplementary Materials

The following supporting information can be downloaded at: https://www.mdpi.com/article/10.3390/diagnostics16132094/s1, Table S1: Overlap between Baseline Comorbidities and 38-item Frailty Components; Table S2. Distribution of Potentially Inappropriate Medication Classes Based on the AGS Beers Criteria.

## Abbreviations

PIM	Potentially Inappropriate Medication
GNRI	Geriatric Nutrition Risk Index
IHD	Ischemic Heart Disease
CVD	Cardiovascular Disease
COPD	Chronic Obstructive Pulmonary Disease
CCI	Charlson Comorbidity Index
mFI	multimorbidity Frailty Index
IQR	Interquartile Ranges
TG	Triglycerides
eGFR	estimated Glomerular Filtration Rate
HTN	Hypertension
Hb	Hemoglobin
HDL	High-Density Lipoprotein
LDL	Low-Density Lipoprotein
GPT	Glutamate Pyruvate Transaminase
WBC	White Blood Cell
OH	Orthostatic Hypotension
BMD	Bone Mineral Density
IL-6	Interleukin-6
CRP	C-reactive Protein
