# Supplementary material for: Association of Potentially Inappropriate Medications and Geriatric Nutritional Risk Index with Frailty in Elderly Patients with Ischemic Heart Disease
Source: Diagnostics (Basel). 2026 Jul 3;16(13):2094. doi: 10.3390/diagnostics16132094 (PMC13361485; doi:10.3390/diagnostics16132094)
Supplement: Supplementary file 1 [file diagnostics-16-02094-s001.zip › diagnostics-4389981-supplementary.pdf]

Table S1: Overlap between Baseline Comorbidities and 38-item Frailty Components.

No.	mFI component	ICD-10-CM code	Overlaps with adjusted covariate?	Adjusted covariate
1	Hypertension	I10	Yes	Hypertension
2	Hypertensive heart disease with/without heart failure	I11	Yes	Hypertension
3	Cerebral infarction	I63	Yes	Cerebrovascular accident
4	Cerebral vascular disease	I67	Yes	Cerebrovascular accident
5	Late effect of cerebrovascular diseases	I69	Yes	Cerebrovascular accident
6	Chronic kidney disease	N18	Yes	Renal disease
7	Chronic obstructive pulmonary disease	J44	Yes	Chronic obstruction pulmonary disease
8	Disorders of electrolyte and fluid balance	E87	No	—
9	Dementia	F03	No	—
10	Sleep disorders and apnea	G47	No	—
11	Disorders of eyelids	H02	No	—
12	Retinopathy and other eye disorders	H35	No	—
13	Glaucoma and ocular hypertension	H40	No	—
14	Vertigo or other disorder of vestibular function	H81	No	—
15	Angina	I20	No	—
16	Atherosclerotic heart disease and chronic ischemic heart disease	I25	No	—
17	Atrial fibrillation and atrial flutter	I48	No	—
18	Cardiac arrhythmia	I49	No	—
19	Heart Failure	I50	No	—
20	Pneumonia	J18	No	—

21	Asthma	J45	No	—
22	Gastric ulcer	K25	No	—
23	Peptic ulcer	K27	No	—
24	Functional dyspepsia	K30	No	—
25	Constipation	K59	No	—
26	Cellulitis	L03	No	—
27	Dermatitis	L30	No	—
28	Gout	M10	No	—
29	Polyosteoarthritis	M15	No	—
30	Osteoarthritis	M19	No	—
31	Spinal stenosis and spondylopathy	M48	No	—
32	Osteoporosis	M81	No	—
33	Urinary tract infection	N39	No	—
34	Enlarged and nodular prostate	N40	No	—
35	Cough	R05	No	—
36	Abdominal pain	R10	No	—
37	Dizziness and giddiness	R42	No	—
38	Presence of functional implant	Z96	No	—

Table S2. Distribution of Potentially Inappropriate Medication Classes Based on the AGS Beers Criteria

PIM Class	Included ATC Codes / Medication Subclasses	Prescription records, *n* (%)	Patients exposed, *n* / 1966 PIM users (%)	Patients exposed, *n* / 3320 total cohort (%)
**Sedative-Hypnotics**	N05BA, N05BB, N05CD, N05CF, N03AE01<br>Benzodiazepines, Z-drugs, Clonazepam	3249 (28.06%)	924 (47.00%)	924 (27.83%)
**Proton Pump Inhibitors (PPIs)**	A02BC03, A02BC05, A02BC06<br>Lansoprazole, Esomeprazole, Dexlansoprazole	3032 (26.18%)	910 (46.29%)	910 (27.41%)
**Cardiovascular Drugs**	B01AC07, C02CA04, C01BD01, C01AA05, C08CA05, G04CA03, C01BD07, C02AC01<br>Dipyridamole, Alpha-blockers, Amiodarone, Digoxin, Nifedipine, Dronedarone, Clonidine	2023 (17.47%)	626 (31.84%)	626 (18.86%)
**Highly Anticholinergic Drugs**	A03, G04BD, N06AA, R06, N04AA01<br>GI/Urinary antispasmodics, TCAs, 1st-Gen Antihistamines, Trihexyphenidyl	1160 (10.02%)	386 (19.63%)	386 (11.63%)
**Pain & Musculoskeletal Drugs**	M01, M03, N02<br>NSAIDs, Skeletal muscle relaxants, Opioids including Tramadol/Meperidine	925 (7.99%)	329 (16.73%)	329 (9.91%)
**Antipsychotics**	N05AB, N05AD, N05AE, N05AH, N05AL, N05AX<br>Prochlorperazine, Haloperidol, Lurasidone, Olanzapine, Quetiapine, Sulpiride, Risperidone, Aripiprazole, Brexpiprazole	599 (5.17%)	197 (10.02%)	197 (5.93%)
**Endocrine System Drugs**	A10BB12, H01BA02, G03, L02AB01<br>Sulfonylureas [Glimepiride], Desmopressin, Estrogens/Androgens, Megestrol	520 (4.49%)	162 (8.24%)	162 (4.88%)
**Other Drugs**	C04AE01, N03AA02, N06AB05, A02BC08<br>Ergoloid mesylates, Phenobarbital,	72 (0.62%)	25 (1.27%)	25 (0.75%)

	Paroxetine, Vonoprazan			
**Total**		11580 (100%)	-	-

Note: All patients in this analysis used at least one PIM. Counts reflect the total number of prescription records for each medication class.
